# Determinants of racial/ethnic differences in blood pressure management among hypertensive patients

**DOI:** 10.1186/1471-2261-5-16

**Published:** 2005-06-22

**Authors:** LeRoi S Hicks, Shimon Shaykevich, David W Bates, John Z Ayanian

**Affiliations:** 1 Division of General Internal Medicine, Brigham and Women's Hospital and Harvard Medical School, Boston, MA, USA; 2 Department of Health Care Policy, Harvard Medical School, Boston, MA, USA; 3 Brigham and Women's-Faulkner Hospitalist Program, Brigham and Women's Hospital, Boston, MA, USA

## Abstract

**Background:**

Prior literature has shown that racial/ethnic minorities with hypertension may receive less aggressive treatment for their high blood pressure. However, to date there are few data available regarding the confounders of racial/ethnic disparities in the intensity of hypertension treatment.

**Methods:**

We reviewed the medical records of 1,205 patients who had a minimum of two hypertension-related outpatient visits to 12 general internal medicine clinics during 7/1/01-6/30/02. Using logistic regression, we determined the odds of having therapy intensified by patient race/ethnicity after adjustment for clinical characteristics.

**Results:**

Blacks (81.9%) and Whites (80.3%) were more likely than Latinos (71.5%) to have therapy intensified (P = 0.03). After adjustment for racial differences in the number of outpatient visits and presence of diabetes, there were no racial differences in rates of intensification.

**Conclusion:**

We found that racial/ethnic differences in therapy intensification were largely accounted for by differences in frequency of clinic visits and in the prevalence of diabetes. Given the higher rates of diabetes and hypertension related mortality among Hispanics in the U.S., future interventions to reduce disparities in cardiovascular outcomes should increase physician awareness of the need to intensify drug therapy more agressively in patients without waiting for multiple clinic visits, and should remind providers to treat hypertension more aggressively among diabetic patients.

## Background

Hypertension is among the most prevalent chronic diseases in the United States [1]. Despite the availability of effective medications and well-published guidelines for the treatment of hypertension [1-4], the majority (approximately 75%) of hypertension in the United States remains poorly controlled [5].

Hypertension is particularly burdensome among racial/ethnic minority groups and hypertension-related cardiovascular disease has been shown to be the greatest contributor to previously documented racial differences in mortality [5-12]. Although higher rates of hypertension control and a reduction in racial differences in outcomes from hypertension may be obtained by increasing providers' aggressiveness in intensifying therapy when indicated [2,12], several studies have demonstrated that providers often allow their patients to remain poorly controlled [12,13].

We previously examined the association of patients' race/ethnicity with processes of hypertension care [13]. We found that, among a cohort of hypertension patients with repeatedly elevated blood pressures, Hispanics were significantly less likely to have therapy intensified and were more likely to have uncontrolled blood pressure (BP) than were other racial and ethnic groups. In an effort to identify potential targets for interventions to improve hypertension care among our patients, we further examined which patient and provider characteristics may explain racial differences in rates of therapy intensification.

## Methods

### Study sample and procedures

To determine which patient-centered characteristics are associated with providers intensifying drug therapy for hypertension and whether provider experience is related to differences in intensification of therapy, we studied a random subset of 1,205 patients who had a minimum of two hypertension-related outpatient visits to one of twelve general medicine clinics in community health centers and community practices affiliated with a large urban academic medical center from July 1, 2001 through June 30, 2002 (totaling 3,257 visits). To determine hypertension-related visits, we reviewed the electronic medical record (EMR) for all clinic visits with a primary or secondary diagnostic code of hypertension (HTN) (ICD-9 401- 401.9, 405- 405.99). The study protocol was approved by the Human Studies Committee at the Brigham and Women's Hospital.

### Medical record and administrative data

We hired three abstractors who were trained by the primary investigator to review the EMR of each patient in our study sample, each abstractor reviewed records for approximately 400 individual patients. From each hypertension-related visit note in the EMR they collected the following data: patient race/ethnicity, name of the patient's primary provider, patient age at time of initial study period visit, sex, primary insurer at time of initial study period visit, presence of comorbid disease (diabetes, congestive heart failure (CHF), coronary artery disease (CAD), or renal failure) listed on the patient problem list, BP control (defined using cut point of <130/85 for diabetic or renal failure patients and <140/90 for all others), and any changes made to antihypertensive drug therapy during visit (decrease or discontinuation of drug, change to another class of drug, or increase of drug dose or addition of new drug).

The EMR of each patient contains racial/ethnic data obtained via self-report the first time each patient registers in his/her clinic that then is classified by the data entry clerk as Hispanic ethnicity or not and race is then classified as Black, White, other, or unknown. Hence, it is impossible to know the country of origin of our Hispanic participants and we could not determine the exact racial/ethnic mix of patients classified as "other" or "unknown." For these reasons, we excluded patients who's EMR listed their racial/ethnic classification as "other" (N = 25) or "unknown" (N = 67) and we limited our analyses to White patients (who identify themselves as non-Hispanic), Black patients (who identify themselves as non-Hispanic), and self-identified Hispanic patients.

From the medical center's administrative database, we abstracted each provider's level of experience (intern, resident, or attending). Patient zip code was also obtained and linked to 2000 U.S. Census data to obtain the median annual household income in each patient's zip code.

### Intensity of therapy

**Figure 1:**
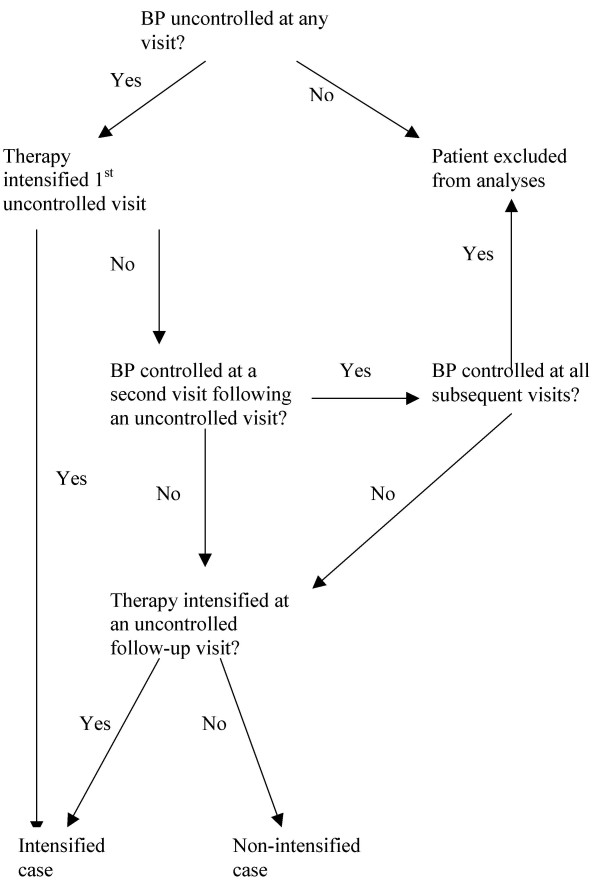
Flow diagram of algorithm for determining an "intensified case." Inter-rater reliability was high (kappa 0.90). We identified a total of 782 cases as either an "intensified case (N = 600) or a "non-intensified case" (N = 157).

Our methods for identifying intensified cases among our cohort have been previously described [13]. Because we were examining the quality of care delivered to hypertension patients prior to July of 2002, approximately one year before the release of the Seventh Report of the Joint National Committee on Prevention, Detection, Evaluation and Treatment of High Blood Pressure (JNC VII) [14], we used JNC VI definitions for blood pressure control in order to examine whether therapy was appropriately intensified. We classified each visit into two categories, intensified visits (an increase in intensity of drug therapy) versus non-intensified visits (a decrease or no change in intensity of drug therapy) according to previously published definitions of changes in medications [2]. We developed an algorithm to determine whether a patient received at least one increase in drug therapy (an increase in drug dose or addition of new medication) in response to a repeatedly elevated BP during the study period (Figure 1). Each patient with fewer than two visits with an elevated BP (N = 356) was excluded from the algorithm. Each patient with an uncontrolled BP at more than one visit was identified as either an intensified case (at least one drug increase) or a non-intensified case (no drug increases). Each reviewer examined a subset of 30 records; we then tested for inter-rater reliability and found excellent agreement among reviewers (kappa = 0.90).

Our intensification algorithm is potentially limited because it does not account for differences in the number of times medication is increased in comparison to the number of uncontrolled BP visits for each patient. To address this issue, we created a secondary measure of intensification by calculating the proportion of times each patient had their therapy increased when their BP was uncontrolled (0 representing no increases and 1 representing an increase in therapy with every uncontrolled visit). We then determined whether the two intensification measures were correlated in order to further validate our algorithm and found that the two measures were highly correlated (coefficient 0.71, P <0.001). Because our algorithm-derived method has the additional advantage of using more than a single visit's BP in determining whether therapy intensification is indicated, we used this measure of intensification for all subsequent analyses.

### Data analysis

We compared patients' demographic, clinical, and provider's characteristics by patient race/ethnicity (Table 1) using chi-squared tests for categorical and Student's t-test for continuous variables. We also estimated the association of being an intensified case with patients' demographic, clinical, and provider's characteristics (Table 2). We report two-tailed P values with statistical significance set at P≤0.05 for all analyses.

**Table 1:** Demographic and clinical characteristics of population by race and ethnicity

**Variable**	**White (N = 304)**	**Black (N = 309)**	**Hispanic (N = 144)**	P value*
Men (%)	27.3	24.3	35.4	0.05
Aged <65 years (%)	54.6	65.1	66.7	0.009
Annual household income^†^:				<0.001
= $35,500	11.9	37.8	29.4	
$35,501–$43,140	11.9	40.8	21.0	
$43,141–$55,365	24.7	15.5	42.7	
> $55,365	51.6	5.9	7.0	
Insurance:				<0.001
Private/Medicare (%)	87.7	69.1	40.3	
Medicaid (%)	4.3	17.6	28.5	
Free care/Self-pay (%)	8.0	13.4	31.3	
Mean number of hypertension visits	2.8	3.0	2.8	0.06
Diabetic (%)	14.5	27.5	27.8	<0.001
Coronary artery disease (%)	5.3	4.2	2.1	0.02
Provider experience (%)^‡^:				<0.001
Intern	2.1	9.3	10.1	
Resident	4.8	23.0	10.1	
Attending	93.2	67.8	79.8	

*Using chi-square tests for categorical variables and ANOVA for mean number of hypertension visits.

^†^N = 734 for those patients with zip code available.

^‡^N = 691 for those patients with provider information available.

**Table 2:** Demographic and clinical characteristics of population by intensification of therapy

**Variable**	**Non-Intensified Case (N = 157)**	**Intensified Case (N = 600)**	P value*
Patient race/ethnicity (%):			0.03
White	19.7	80.3	
Black	18.1	81.9	
Hispanic	28.5	71.5	
Men (%)	29.1	27.7	0.74
Age:			0.10
<65 years	18.8	81.2	
≥65 years	23.8	76.2	
Annual household income^†^:			0.93
≤$35,500	20.4	79.6	
$35,501–$43,140	19.7	80.3	
$43,141–$55,365	22.4	77.6	
> $55,365	20.5	79.5	
Insurance:			0.15
Private/Medicare (%)	19.5	80.5	
Medicaid (%)	27.8	72.2	
Free care/Self-pay (%)	20.0	80.0	
Mean number of hypertension visits	2.6	3.0	<0.001
Diabetic (%):			0.01
Yes	27.8	72.2	
No	18.7	81.3	
Coronary artery disease (%):			0.24
Yes	12.5	87.5	
No	21.1	78.9	
Provider training level (%)^‡^:			0.91
Intern	22.7	77.3	
Resident	21.4	78.6	
Attending	20.3	79.7	

*Using chi-square tests for categorical variables and Student's *t*-test to compare mean hypertension visits.

^†^N = 734 for those patients with zip code available.

^‡^N = 691 for those patients with provider information available.

Using logistic regression, we assessed whether race/ethnicity was associated with intensification of therapy after adjusting for all measured confounders. Data were available on every variable for 685 of the 757 patients (90.5%) for multivariable analyses. We report adjusted odds ratios with 95% confidence intervals for the intensified cases. In secondary analyses, we included interaction terms for patient race/ethnicity and provider experience level to determine whether racial/ethnic differences in intensification differed by provider experience level. All non-significant interaction terms were removed from the final model. All models were estimated using SUDAAN statistical software to adjust for within clinic correlation of visits [15].

## Results

### Patient, clinical, and provider characteristics

Of the 757 patients, 304 (40%) were White, 309 (41%) were Black, and 144 (19%) were Hispanic (Table 1). The majority of patients in our cohort were women (72%) and most were either privately insured or had Medicare (71%). There were 169 (22%) patients in our cohort with diabetes. We were able to determine the primary provider for 691 patients, of who 44 (6.4%) received care from an intern, 89 (12.9%) received care from a resident, and 558 (80.8%) received care from an attending. Demographic and clinical differences between patients' racial/ethnic groups are presented in Table 1.

### Intensification of therapy

Of the 757 patients with multiple visits whose BP was uncontrolled at two or more visits, there were 600 (79.3%) who had their medications intensified. Hispanic patients were significantly less likely to have their medications intensified than White and Black patients (Table 2). Diabetic patients and those with fewer visits during the study period were also less likely to have their medications intensified.

After adjustment for age, insurance status, number of hypertension-related visits, diabetes, and physician experience, there were no remaining racial/ethnic differences in rates of intensification (Table 3). Compared to patients aged = 65 years, younger patients were more likely to have their therapy intensified, and the odds of having therapy intensified increased with each visit. There were no significant interactions between race/ethnicity and provider experience in the multivariable model.

**Table 3:** Adjusted odds ratios* [95% C.I.] of being an intensified case

**Variable**	**Odds Ratios of being Intensified Case**	**95 % Confidence interval**
Black^†^	1.09	[0.71–1.67]
Hispanic^†^	0.78	[0.58–1.06]
Age < 65 years^‡^	1.61	[1.38–1.88]
Medicaid^§^	0.76	[0.57–1.02]
Free care or self pay^§^	1.22	[0.73–2.03]
Number of hypertension visits^||^	1.37	[1.17–1.61]
Diabetes**	0.61	[0.34–1.08]
Intern provider^††^	0.86	[0.39–1.88]
Resident provider^††^	0.89	[0.55–1.46]

* Adjusted for patient race/ethnicity, age, insurance status, number of hypertension-related visits, diabetes, and physician experience.

^†^Whites use as reference group

^‡^Aged ≥ 65 years used as reference group

^§^Privately insured or Medicare used as reference group

^||^Represents increase in odds with each additional visit

**No diabetes used as reference group

^††^Attending provider used as reference group

## Discussion

In our prior work, we found that Hispanic patients were more likely to receive the JNC recommended antihypertensive drug class then Whites, however, Hispanics were also significantly less likely than Whites to have their therapy appropriately intensified in response to an uncontrolled BP [13]. Furthermore, we found appropriate intensification of anti-hypertensive therapy was associated with subsequent BP control for all racial/ethnic groups, suggesting that the poorer rates of BP control among Hispanics in our prior study may have been due to significantly lower rates of antihypertensive medication intensification [13]. In this study, we found that these previously noted racial/ethnic disparities in rates of antihypertensive therapy intensification may be due to differences in visit patterns among patients and in physicians' aggressiveness in managing BP in diabetic patients, suggesting that racial/ethnic differences in disease severity are likely determinants of unequal treatment of uncontrolled hypertension.

There is substantial literature, that suggests that racial/ethnic minority groups are less likely to have their antihypertensive therapy appropriately intensified [16-19]. However, these studies were limited because the investigators were not able to assess practice patterns such as the frequency with which individual anti-hypertensive drugs were intensified in response to uncontrolled BP. We found that Hispanic patients in our cohort were also less likely to have their anti-hypertensive medications intensified at least once in response to repeatedly uncontrolled BP than were other racial/ethnic groups and our findings also expand beyond documenting racial/ethnic disparities in aggressiveness of therapy by determining the roles racial/ethnic differences in clinic utilization among patients and racial/ethnic differences in the prevalence of diabetes play in confounding differences in providers' aggressiveness in treating hypertension.

An important national priority in health care is the elimination of racial/ethnic disparities in healthcare; however, a better understanding of the determinants of disparities is needed to address this issue [20,21]. Our finding that higher rates of diabetes among Hispanics in our cohort play a major role in the insufficient management of their uncontrolled BP is of particular concern given that Hispanics suffer a disproportionately larger burden from hypertension and diabetes compared to Whites; and that Hispanics are at a higher risk of having hypertension and diabetes, are less likely to be aware that they are hypertensive, are more likely to have target organ damage, and have significantly higher age-adjusted diabetes and hypertension-related mortality compared to whites in the U.S [5,6,22,23]. Our findings are supported by several studies that have documented lower rates of BP control among diabetic patients [24,25], and others showing that providers often do not attain adequate BP control for patients, even after multiple opportunities to do so [2,24].

Our study has several limitations. We were unable to collect measures of patient adherence to prescribed therapy from the EMR. Providers may not want to intensify therapy at the same rate for patients they know are less compliant with therapy, although it is hard to identify these patients. We were also unable to determine English proficiency of each patient from the medical records. When a language barrier exists providers may be less likely to intensify therapy.

We examined disparities in quality of hypertension care, as measured by intensification of therapy, among a cohort of patients and physicians during 2001–2002 using the established guidelines available during that time period, the JNC VI, as the reference for our analyses. In comparison to JNC VI, the JNC VII guidelines recommend much more aggressive management of hypertension both in terms of the accepted level of BP control (130/80 for diabetic and renal failure patients and 140/90 for all others) and the recommendations to providers to intensify antihypertensive therapy more rapidly when BP is uncontrolled [14]. However, since the JNC VII guidelines were not available until 2003, it would have been impossible for providers in our study sample to have been fully aware of them and it would have been misleading to assess the quality of hypertension care among our cohort using quality guidelines that were not in existence during that time. By using the less aggressive guidelines that were in existence during the study period, we may have underestimated the gap in rates of intensification between racial/ethnic groups and between patients with diabetes and those without.

Lastly, we examined patients who received their care at primary care practices affiliated with a single large urban teaching hospital, and although there was substantial socioeconomic diversity in our sample our results may not be generalizable to smaller, rural, or non-teaching hospitals.

## Conclusion

We found significant racial/ethnic differences in intensification of drug therapy, and that these differences were largely accounted for by differences in frequency of clinic visits and in the prevalence of diabetes. Future interventions should focus on increasing physician awareness of the need to intensify drug therapy more, particulary among Hispanic patients, and on encouraging providers to treat hypertension more intensively in diabetic patients.

## Competing interests

The author(s) declare that they have no competing interests.

## Authors' contributions

LH conceived of the study, and participated in its design and coordination and helped to draft the manuscript. SS participated in the design of the study, performed the statistical analysis, and helped to draft the manuscript. DWB and JZA participated in the design of the study, and helped to draft the manuscript.

## Pre-publication history

The pre-publication history for this paper can be accessed here:


